# Gadopentetate dimeglumine extravasation resulting in cutaneous ulceration: a case report

**DOI:** 10.1186/s13256-026-06017-8

**Published:** 2026-04-15

**Authors:** Wenyan Zhang, Bingyan Wang, Xianrong Kong, Qin Li

**Affiliations:** 1 Department of Radiology, Tongji Hospital, Tongji Medical College, Huazhong University of Science and Technology, Wuhan, China; 2 Department of Nursing, Tongji Hospital, Tongji Medical College, Huazhong University of Science and Technology, Wuhan, China

**Keywords:** Gadolinium-based contrast agents (GBCAs), Gadopentetate dimeglumine, Extravasation, Cutaneous ulceration

## Abstract

**Background:**

Extravasation of gadolinium-based contrast agents (GBCAs) during magnetic resonance imaging is a rare adverse event, with most cases resolving without significant complications. However, severe tissue injury following GBCA extravasation remains extremely rare and incompletely characterized in the existing literature.

**Case presentation:**

A 58-year-old female (Chinese, Asian) with hepatocellular carcinoma and ongoing chemotherapy experienced gadopentetate dimeglumine extravasation during an abdominal magnetic resonance imaging (MRI) examination. Despite standard initial management—including immediate catheter removal, ice application, limb elevation, and topical heparinoid cream administration—the patient developed a tension blister within four hours, followed by progressive tissue necrosis and a deep cutaneous ulcer. The ulceration was further complicated by secondary infection, likely secondary to inappropriate self-management of the blister. The patient underwent a prolonged wound care regimen, culminating in complete re-epithelialization after four weeks, with no residual functional impairment.

**Conclusion:**

This case underscores the potential severity of cutaneous complications resulting from GBCA extravasation, especially in high-risk patients with chemotherapy-induced vascular vulnerability or diminished tissue integrity. It emphasizes the critical importance of vigilant post-extravasation monitoring, proactive patient education to prevent inappropriate self-management of the injury, and the implementation of structured wound care protocols. Future studies are warranted to establish evidence-based preventive strategies and therapeutic guidelines for GBCA-related extravasation injuries.

## Introduction

Gadolinium-based contrast agents (GBCAs) are the most widely used contrast enhancers in magnetic resonance imaging (MRI). By altering the relaxation rates of water protons, GBCAs significantly improve lesion detection and tissue contrast in MRI, playing an indispensable role in tumor diagnosis, vascular imaging, and neurological disease evaluation [1, 2]. Clinical data indicate that over 500 million doses have been administered globally, underscoring their critical importance in diagnosing various diseases [3]. During examinations, GBCAs are typically administered intravenously via a power injector at rates ranging from 1.0 to 3.0 mL/s, depending on the specific imaging requirements. Extravasation of GBCAs refers to the adverse event in which the contrast agent leaks from the vein into surrounding soft tissues during injection, leading to localized swelling, pain, and impaired image enhancement on MRI. The current research on contrast extravasation has largely focused on iodinated agents used in contrast-enhanced CT, while extravasation of GBCAs has been far less frequently reported. Severe tissue injury resulting from GBCA extravasation remains extremely rare and is inadequately documented in the literature.

The reported extravasation rate for GBCAs is 0.045%, nearly six times lower than that of iodinated contrast agents [4]. Elderly, female, and hospitalized patients are at higher risk [4]. From January 2023 to December 2024, our institution performed 98,466 enhanced MRI examinations, with 26 cases of extravasation (0.026%). Among these, one patient developed tissue ulceration at the extravasation site, while the others recovered without complications. This case of tissue ulceration is presented herein.

## Case presentation

A 58-year-old female (Chinese, Asian) with a height of 160 cm and weight of 74 kg, was clinically diagnosed with malignant liver tumor and had undergone comprehensive treatment for over seven months. Her medical history included hepatitis C for more than ten years, left arm osteomyelitis surgery in her teens, hypertension, and pulmonary hypertension. She had no history of allergies. Positive laboratory findings included: total protein 68 g/L (reference range: 66–87 g/L), albumin 39.8 g/L (reference range: 40–55 g/L), eGFR 79.8 (reference value: > 90), and platelet count 123 × 10^9^/L (reference range: 125–350 × 10^9^/L). She was receiving recombinant human thrombopoietin injection at the time of examination.

**Fig. 1:**
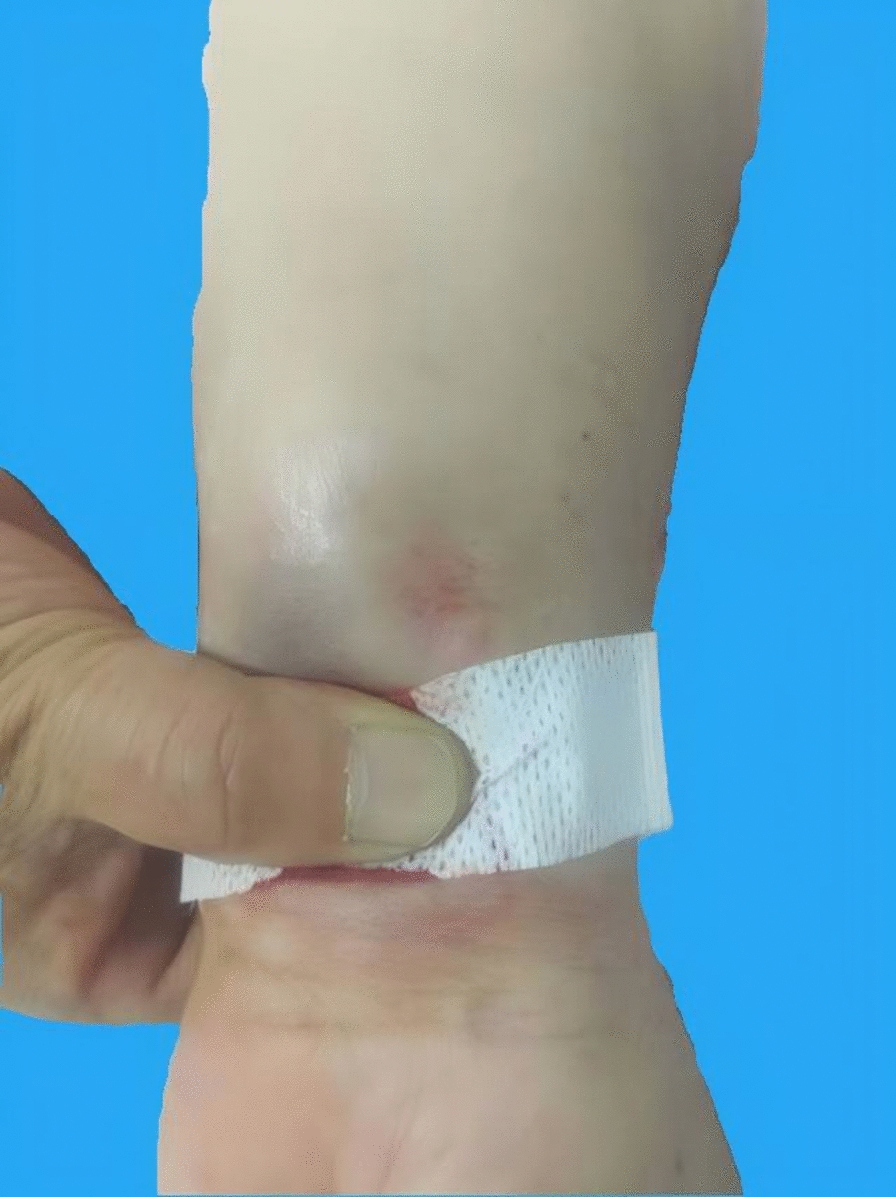
Local manifestations of gadopentetate dimeglumine extravasation (June 29, 2024)

On June 29, 2024, the patient presented for an MRI examination of the liver, including plain scan, diffusion-weighted imaging, and perfusion imaging. A radiology nurse inserted a 22G Safety IV Catheter into the medial left forearm. After patency was confirmed, the catheter was connected to a power injector. The contrast agent used was gadopentetate dimeglumine injection, with a volume of 15 mL administered at a rate of 2.5 mL/s. The injection proceeded smoothly, and arterial phase imaging demonstrated ideal enhancement. After the examination, the nurse observed mild extravasation above the puncture site, with a swollen area of approximately 3 × 5 cm (Fig. 1). The intravenous catheter was immediately removed, manual compression was applied for hemostasis, and local ice packs administered. The affected limb was elevated above heart level. After 30 min of ice application, the swelling had decreased slightly. Heparinoid cream was topically applied with gentle massage to promote absorption.

**Fig. 2:**
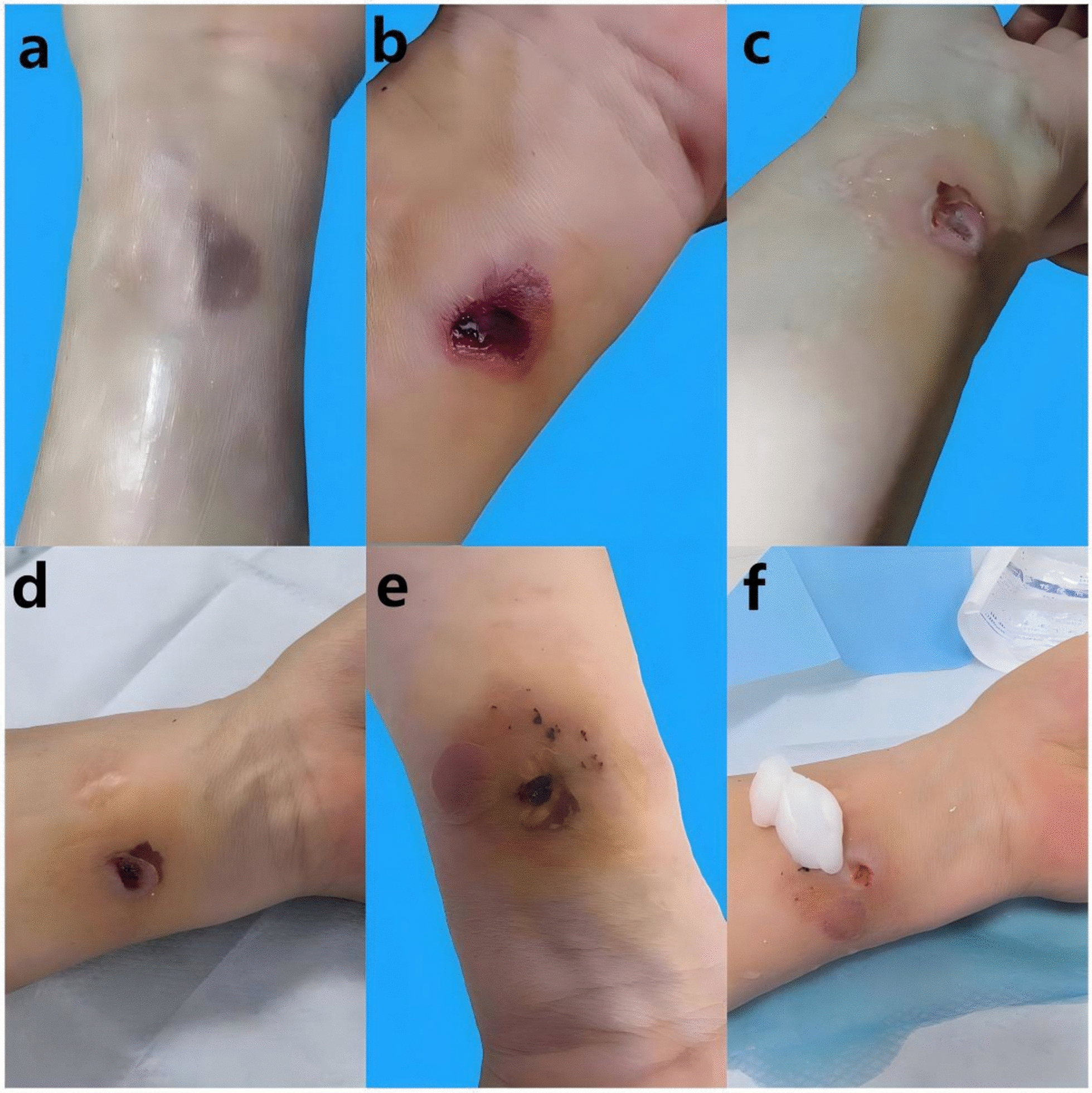
Local manifestations of gadopentetate dimeglumine extravasation: **a** July 2; **b** July 4; **c** July 9; **d** July 10; **e** July 13; **f** July 15

**Fig. 3:**
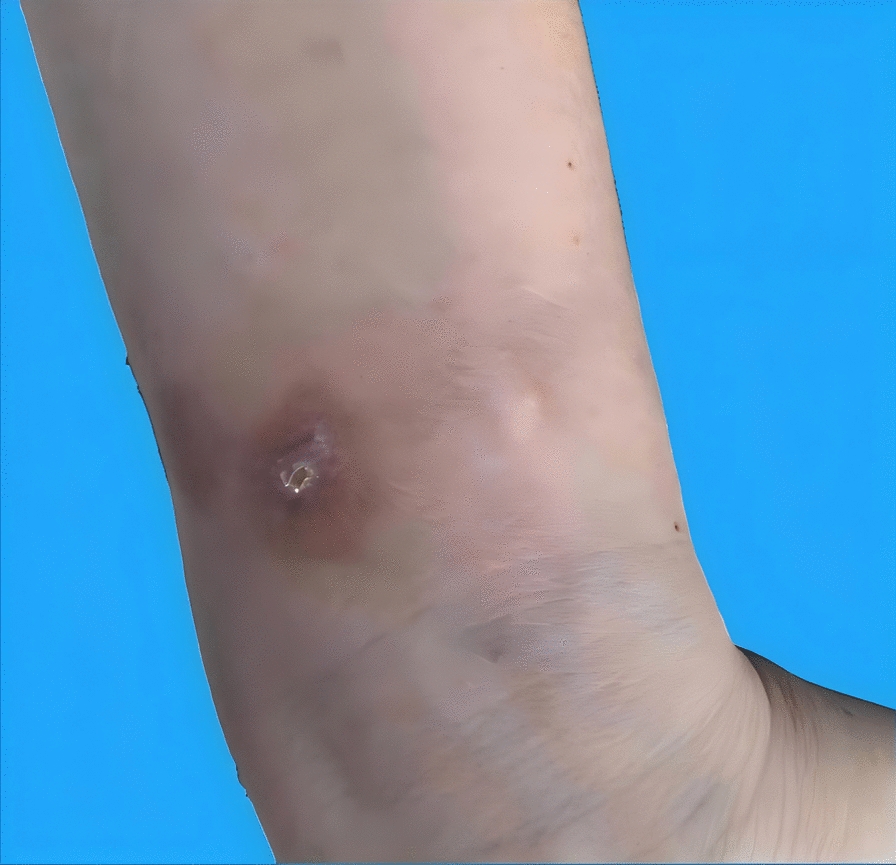
Local manifestations of gadopentetate dimeglumine extravasation (July 31)

After onsite management and one hour of observation, the patient was instructed to continue ice application (every 2 h) and limb elevation at home. She was advised to seek immediate medical attention if symptoms worsened, such as blister formation, increased swelling, intensified pain, or sensory abnormalities. The patient fully understood the measures suggested by the nurse and promised to continue to implement them after returning home. The nurse maintained continuous contact with the patient via a social messaging application, and was available to address any questions or concerns at all times. Within four hours, a tension blister developed and was managed conservatively. However, the patient punctured the blister herself without medical supervision on June 30 and applied methylrosanilinium chloride, leading to progressive tissue darkening and expansion of the affected area. By July 2, the lesion showed dark purple discoloration with blistering (1 × 2 cm) (Fig. 2a), prompting referral to a community clinic, where the wound was disinfected with povidone-iodine and topical mupirocin ointment was administered. The wound evolved into a central eschar surrounded by erythema (Fig. 2b), and on July 9, debridement was performed for a ∼ 2 mm deep ulcer with gray-white necrotic base (Fig. 2c). The patient was referred to a tertiary wound clinic on July 10 for regular dressing changes. Despite temporary worsening with renewed blistering and inflammation on July 13 (Fig. 2e), the ulcer showed robust granulation tissue by July 15 (Fig. 2f) and achieved complete healing without functional impairment by July 31 (Fig. 3). Table 1 documents the detailed clinical course and corresponding interventions.

**Table 1:** The sequence of events and interventions

Date	Local tissue condition	Interventions
June 29	Extravasation of gadopentetate dimeglumine; swollen area ~ 3 × 5 cm; skin ecchymosis	IV catheter removed; manual compression for hemostasis; ice pack application and limb elevation for 30 min; topical heparinoid cream application
June 29	Blister (soybean-sized) appeared at extravasation site 4 h	Blister kept intact; heparinoid cream applied (avoiding wound); fresh potato slice compress on swollen area
June 30	Patient self-punctured the blister	Topical application of methylrosanilinium chloride solution; wound kept dry
July 2	Dark purple tissue with blister; area 1 × 2 cm	Visited local community clinic; wound disinfected with povidone-iodine; topical mupirocin ointment applied
July 4	Central scabbing; surrounding dark red area; 2 × 3 cm	Wound kept dry; povidone-iodine disinfection; topical mupirocin ointment continued
July 9	The eschar detached, revealing a depressed ulcer approximately 2 mm deep with central gray-white necrotic tissue	Debridement and dressing change at community clinic
July 10	Central ulcer area scabbed; surrounding pink granulation tissue observed	Referred to tertiary hospital wound clinic; dressing change every two days with observation
July 13	Redness and inflammation around ulcer; area 4 × 5 cm; new blister ~ 1.5 cm in diameter	Wound disinfected; blister fluid aspirated; continued dressing changes and observation
July 15	Ulcer healing well; granulation tissue visible after eschar removal; surrounding inflammation improved	Continued dressing changes and observation
July 31	Ulcer completely healed; no functional impairment of the limb	

## Discussion

Gadolinium-based contrast agents (GBCAs) are the most commonly used paramagnetic contrast agents in MRI. Their core component is the gadolinium ion (Gd^3^⁺). Based on the structure, they are classified as linear or macrocyclic, with the latter exhibiting higher stability [5, 6]. As essential contrast agents for MRI, GBCAs have raised primary safety concerns regarding nephrogenic systemic fibrosis (NSF) [7, 8], gadolinium deposition [3, 9], and potential toxicity [10]. This has prompted more cautious selection of agent types and accelerated innovation in alternative techniques [5, 7, 11]. Although GBCA extravasation rarely results in severe adverse outcomes, the resulting tissue swelling, pain, and temporary limb functional limitations are clinically significant, causing psychological distress and delays in treatment plans. The ulceration observed in this case suggests that GBCA extravasation can cause significant harm and warrants attention.

The patient was a middle-aged female with malignant liver tumor undergoing intravenous chemotherapy for seven months. Chemotherapy-induced peripheral venous damage and age-related increased venous fragility were likely the primary causes of extravasation. Coagulation tests revealed thrombocytopenia, and she was receiving recombinant human thrombopoietin therapy. This explains the ecchymosis observed at the extravasation site (Fig. 1), likely due to inadequate hemostasis following vessel rupture during high-pressure injection. The appearance of a tension blister four hours post-extravasation was not observed in any of the other 25 cases of GBCA extravasation at our center in the past two years, nor has it been commonly reported elsewhere [12]. The blister formation in this patient may be explained by three factors: First, studies analyzing MRI characteristics after subcutaneous GBCA extravasation have shown local tissue signal abnormalities indicative of edema and inflammatory changes [12]. The patient’s pre-existing hypoalbuminemia may have contributed to underlying tissue edema, exacerbating swelling and tissue tension. Second, the agent used, gadopentetate dimeglumine, is a linear chelate with gadopentetic acid diglumine as its primary constituent (gadolinium diethylenetriaminepentaacetic acid bis-methylglucamine). Its molecular formula is C₁₄H₂₀GdN₃O₁₀·2C₇H₁₇NO₅, molecular weight 938.01, and osmolality approximately 1940 mOsm/kg, significantly higher than human plasma osmolality (290 mOsm/kg). This means that upon subcutaneous extravasation, besides drug-induced swelling, it draws significant interstitial fluid into the subcutaneous space to achieve osmotic equilibrium, further aggravating swelling [10, 13]. Additionally, the molecular weight of gadopentetate dimeglumine (938.01 Da) exceeds the 800 Da threshold for rapid transmembrane absorption. Compounds with a molecular weight above 800 are primarily absorbed via the lymphatic system, resulting in slower clearance and prolonged soft tissue swelling [14, 15]. Third, the extravasation site was located on the medial forearm near the wrist, where subcutaneous tissue is thin, offering poor buffering capacity and absorption, predisposing the site to increased tissue tension.

The tissue breakdown and ulcer formation were likely related to wound infection, though the influence of the patient’s arm osteomyelitis surgery over 40 years prior remains uncertain. According to general wound management principles, small blisters may not require drainage, while large blisters should be aspirated under aseptic conditions after disinfection, with the epidermis preserved as a natural barrier against bacterial infection [13]. In this case, the patient disregarded nursing instructions and punctured the blister herself without medical supervision, and applied Methylrosanilinium Chloride Solution from her personal supply—a medication not recommended for such wounds. This likely triggered the wound deterioration. Additionally, the patient was in an intermittent phase of chemotherapy for liver cancer, with relatively compromised immunity, which was also a risk factor for wound worsening.

Regarding contrast extravasation, although robust clinical trial evidence is lacking, currently accepted management includes ice packs and elevation of the affected limb to reduce swelling and promote absorption [16–18], which is often effective for mild cases. These are the basic nursing measures adopted at our center for contrast extravasation. Additionally, heparinoid cream is commonly used due to its ability to promote blood circulation and improve phlebitis. In this case, despite active management and observation, blistering and ulceration still occurred. After a month of treatment and observation, the wound eventually healed without sensory or functional deficits. However, the patient experienced significant psychological burden, mobility limitations, and additional medical costs. This case highlights the need for increased vigilance in cases involving patients with multiple comorbidities, ongoing chemotherapy, or extravasation at sites with thin subcutaneous tissue (e.g., dorsum of hand, wrist). Particularly when tension blisters appear, decisions regarding aspiration should be based on the clinical situation, ensuring strict aseptic technique to prevent secondary infection.

This study presents a rare case of cutaneous ulceration resulting from gadopentetate dimeglumine extravasation, which offers a cautionary insight and underscores the need for heightened clinical awareness. However, several limitations must be acknowledged. First, as the patient was managed on an outpatient basis, wound monitoring and some aspects of the patient’s wound care were self-performed under remote nursing guidance. Second, constrained by logistical limitations and patient preference, no imaging studies were performed to assess the extravasation site, nor were laboratory tests, such as wound culture, conducted. Future studies should incorporate more stringent follow-up protocols for similar cases, including systematic radiologic evaluation and microbiological analysis of wounds, to better elucidate the progression of tissue injury and facilitate targeted therapeutic management.

## Conclusion

This case report analyzed the potential risk factors and management strategies for cutaneous ulceration secondary to GBCA extravasation. Existing literature primarily focuses on iodinated contrast extravasation, with limited in-depth exploration of risk factors, injury mechanisms, and management strategies for GBCA extravasation. In addition to concerns regarding NSF, gadolinium deposition, and toxicity, tissue injury following extravasation should be recognized as a critical safety consideration for the clinical use of GBCAs.

## Data Availability

The datasets related to this study are available from the corresponding author upon reasonable request.

## Abbreviations

EGFR: Estimated glomerular filtration rate
GBCA: Gadolinium-based contrast agent
IV: Intravenous
MRI: Magnetic resonance imaging
NSF: Nephrogenic systemic fibrosis

## References

[1] Carbone C, Stoeckle A, Minardi M, Uggeri F, Lattuada L, Minguzzi A, *et al*. Electrochemical method for the design of new possible Gadolinium-based contrast agents. Nanomaterials. 2024;14(24):1979. 39728515 10.3390/nano14241979 PMC11679725

[2] Orts-Arroyo M, Ten-Esteve A, Ginés-Cárdenas S, Castro I, Martí-Bonmatí L, Martínez-Lillo J. A Gadolinium(III) complex based on the thymine nucleobase with properties suitable for magnetic resonance imaging. Int J Mol Sci. 2021;22(9):4586. 33925589 10.3390/ijms22094586 PMC8123898

[3] Davies J, Siebenhandl-Wolff P, Tranquart F, Jones P, Evans P. Gadolinium: pharmacokinetics and toxicity in humans and laboratory animals following contrast agent administration. Arch Toxicol. 2022;96(2):403–29. 34997254 10.1007/s00204-021-03189-8 PMC8837552

[4] Heshmatzadeh Behzadi A, Farooq Z, Newhouse JH, Prince MR. MRI and CT contrast media extravasation: a systematic review. Medicine (Baltimore). 2018;97(9):e0055. 29489663 10.1097/MD.0000000000010055 PMC5851722

[5] Tweedle MF. Alternatives to Gadolinium-based contrast agents. Invest Radiol. 2021;56(1):35–41. 32932378 10.1097/RLI.0000000000000725

[6] Iacobellis F, Di Serafino M, Russo C, Ronza R, Caruso M, Dell’Aversano Orabona G, *et al*. Safe and informed use of gadolinium-based contrast agent in body magnetic resonance imaging: where we were and where we are. J Clin Med. 2024;13(8):2193. 38673466 10.3390/jcm13082193 PMC11051151

[7] Lancelot E, Raynaud JS, Desché P. Current and future MR contrast agents: seeking a better chemical stability and relaxivity for optimal safety and efficacy. Invest Radiol. 2020;55(9):578–88. 32776767 10.1097/RLI.0000000000000684

[8] Lim RP, Hecht EM, Desmond PM. Noncontrast magnetic resonance angiography in the era of nephrogenic systemic fibrosis and Gadolinium deposition. J Comput Assist Tomogr. 2021;45(1):37–51. 32976265 10.1097/RCT.0000000000001074

[9] Henderson IM, Benevidez AD, Mowry CD, Watt J, Bachand GD, Kirk ML, *et al*. Precipitation of Gadolinium from magnetic resonance imaging contrast agents may be the brass tacks of toxicity. Magn Reson Imaging. 2025;119:110383. 40064247 10.1016/j.mri.2025.110383 PMC12088623

[10] Zorigt O, Yasuda H, Nakajima T, Tsushima Y. Concentration-dependent bidirectional modification of evoked synaptic transmission by Gadolinium and adverse effects of Gadolinium-based contrast agent. J Neurosci. 2025;45(17):e1622242025. 40097182 10.1523/JNEUROSCI.1622-24.2025 PMC12019106

[11] Yuan X, Yu H, Wang L, Uddin MA, Ouyang C. Nitroxide radical contrast agents for safe magnetic resonance imaging: progress, challenges, and perspectives. Mater Horiz. 2025;12(6):1726–56. 39757847 10.1039/d4mh00995a

[12] Hama Y, Tate E. MRI findings of iatrogenic extravasation of gadolinium-based contrast agents in patients with cancer. Acta Radiol. 2023;64(4):1439–42. 36221814 10.1177/02841851221129152

[13] Weimer DS, Jones S, Ramadoss T, Milovanovic U, Shoja MM, Schwartz G. Compartment Syndrome Secondary to Calcium Gluconate Extravasation. Cureus. 2023 July 21. https://www.cureus.com/articles/166831-compartment-syndrome-secondary-to-calcium-gluconate-extravasation. Accessed 21 Aug 2025. 10.7759/cureus.42237 PMC10440588 37609086

[14] Zheng F, Hou P, Corpstein CD, Park K, Li T. Multiscale pharmacokinetic modeling of systemic exposure of subcutaneously injected biotherapeutics. J Control Release. 2021;337:407–16. 34324897 10.1016/j.jconrel.2021.07.043

[15] Rahimi E, Aramideh S, Han D, Gomez H, Ardekani AM. Transport and lymphatic uptake of monoclonal antibodies after subcutaneous injection. Microvasc Res. 2022;139:104228. 34547346 10.1016/j.mvr.2021.104228

[16] Stefanos SS, Kiser TH, MacLaren R, Mueller SW, Reynolds PM. Management of noncytotoxic extravasation injuries: a focused update on medications, treatment strategies, and peripheral administration of vasopressors and hypertonic saline. Pharmacother J Hum Pharmacol Drug Ther. 2023;43(4):321–37. 10.1002/phar.2794 36938775

[17] Ding S, Meystre NR, Campeanu C, Gullo G. Contrast media extravasations in patients undergoing computerized tomography scanning: a systematic review and meta-analysis of risk factors and interventions. JBI Database Syst Rev Implement Rep. 2018;16(1):87–116. 10.11124/JBISRIR-2017-003348 PMC5771689 29324560

[18] Bellin MF, Jakobsen JÅ, Tomassin I, Thomsen HS, Morcos SK, Members Of The *Contrast Media Safe. Contrast medium extravasation injury: Guidelines for prevention and management. Eur Radiol. 2002;12(11):2807–12. 12386778 10.1007/s00330-002-1630-9

